# 4501 Statistical Modeling for Predicting Correct Drug Dose in the Presence of Conflicting Dose Information Extracted from Electronic Health Records

**DOI:** 10.1017/cts.2020.185

**Published:** 2020-07-29

**Authors:** Michael Lee Williams, Hannah L Weeks, Cole Beck, Elizabeth McNeer, Leena Choi

**Affiliations:** 1 Vanderbilt University Medical Center

## Abstract

OBJECTIVES/GOALS: Diverse medication-based studies require longitudinal drug dose information. EHRs can provide such data, but multiple mentions of a drug in the same clinical note can yield conflicting dose. We aimed to develop statistical methods which address this challenge by predicting the valid dose in the event that conflicting doses are extracted. METHODS/STUDY POPULATION: We extracted dose information for two test drugs, tacrolimus and lamotrigine, from Vanderbilt EHRs using a natural language processing system, medExtractR, which was developed by our team. A random forest classifier was used to estimate the probability of correctness for each extracted dose on the basis of subject longitudinal dosing patterns and extracted EHR note context. Using this feasibility measure and other features such as a summary of subject dosing history, we developed several statistical models to predict the dose on the basis of the extracted doses. The models developed based on supervised methods included a separate random forest regression, a transition model, and a boosting model. We also considered unsupervised methods and developed a Bayesian hierarchical model. RESULTS/ANTICIPATED RESULTS: We compared model-predicted doses to physician-validated doses to evaluate model performance. A random forest regression model outperformed all proposed models. As this model is a supervised model, its utility would depend on availability of validated dose. Our preliminary result from a Bayesian hierarchical model showed that it can be a promising alternative although performing less optimally. The Bayesian hierarchical model would be especially useful when validated dose data are not available, as it was developed in unsupervised modeling framework and hence does not require validated dose that can be difficult and time consuming to obtain. We evaluated the feasibility of each method for automatic implementation in our drug dosing extraction and processing system we have been developing. DISCUSSION/SIGNIFICANCE OF IMPACT: We will incorporate the developed methods as a part of our complete medication extraction system, which will allow to automatically prepare large longitudinal medication dose datasets for researchers. Availability of such data will enable diverse medication-based studies with drastically reduced barriers to data collection.

